# Bone marrow mesenchymal stem cells-derived exosomal microRNA-193a reduces cisplatin resistance of non-small cell lung cancer cells via targeting LRRC1

**DOI:** 10.1038/s41419-020-02962-4

**Published:** 2020-09-25

**Authors:** Hongbo Wu, Xiaoqian Mu, Lei Liu, Huijuan Wu, Xiufeng Hu, Lijuan Chen, Jie Liu, Yu Mu, Fangfang Yuan, Wenjing Liu, Yanqiu Zhao

**Affiliations:** 1 Department of Internal Medicine, The Affiliated Tumor Hospital of Zhengzhou University, He’nan Cancer Hospital, Zhengzhou, He’nan Province 450008 China; 2 Department of Oncology, West China Hospital of Sichuan University, Chengdu, Sichuan Province 610064 China; 3 Department of Hematology, The Affiliated Tumor Hospital of Zhengzhou University, He’nan Cancer Hospital, Zhengzhou, He’nan Province 450008 China

**Keywords:** Cell biology, Diseases

## Abstract

Exosomes are small endogenous membrane vesicles that can mediate cell communication by transferring genetic materials. Based on that, exosomes have always been discussed as a cargo carrier for microRNA (miRNA) transportation. Accumulating data have reported the inhibitory effects of microRNA-193a (miR-193a) on non-small cell lung cancer (NSCLC) cell progression. However, the mechanisms of miR-193a delivery to cancer cells and miR-193a in exosomes have not been explored clearly in NSCLC. Given that, this work aims to decode exosomal miR-193a in cisplatin (DDP) resistance of NSCLC cells. A549 and H1299 cell lines were screened out and their parent cells and drug-resistant cells were co-cultured with human bone marrow mesenchymal stem cells (BMSCs)-derived exosomes (BMSC-Exo) that had been transfected with miR-193a mimic or si-LRRC1 to detect the colony formation, migration, apoptosis, invasion and proliferation of NSCLC cells. In vivo experiment was conducted to verify the in vitro results. BMSC-Exo with upregulated miR-193a and downregulated LRRC1 suppressed colony formation, invasion, proliferation and migration as well as advanced apoptosis of NSCLC parent cells and drug-resistant cells. BMSC-Exo combined with upregulated miR-193a reduced tumor volume and weight in mice with NSCLC. Functional studies report that BMSC-Exo shuffle miR-193a to suppress the colony formation, invasion, migration, and proliferation as well as advance apoptosis of NSCLC DDP-resistant cells via downregulating LRRC1.

## Background

Lung cancer is the commonest cancer around the world which causes a severe social burden^1^. It is estimated that more than 7,333,000 lung cancer patients are diagnosed annually and the mortality are 6,102,000 in China^2^. As the most common subtype of lung cancer, non-small cell lung cancer (NSCLC) accounts for nearly 80% of lung cancers^3^. According to the statistics, the poor 5-year survival rate NSCLC victims is 21%^4^. The pathogenesis of lung cancer contains living environment, genetic indices and smoking, while the occurrence of lung cancer is connected with gene mediation^5^. Dyspnea, pain, fatigue, anxiety, inappetence, depression, and sleep disorders are all the manifested symptoms in NSCLC^6^. Cisplatin (DDP)-targeted chemotherapy is a general treatment for lung cancer, but chemotherapy resistance and adverse reaction, especially cardiac toxicity have limited its curative effects^7^. Therefore, how to overcome DPP resistance tops an urgency in NSCLC treatment.

Bone marrow-derived mesenchymal stem cells (BMSCs) are pluripotent stromal cells, which are recruited into tumors and contributory to the development of cancer^8^. Exosomes are enclosed vesicles with small membrane which are engaged in intercellular communication. Exosomes load proteins, lipids, and nucleic acids, such as mRNA and microRNA (miRNA), which can be used as biomarkers of cancers^9^. miRNAs are a kind of small non-coding RNAs comprised of 22–25 nucleotides, which are highly implicated in modulating pathophysiologic mechanisms via repressing target gene expression^10^. miRNAs can induce translational inhibition or degradation of its mRNA target, thus constituting a key part of post-transcriptional regulation of mRNA expression. In addition, exosomal miRNA is also closely related to cancer progression^11^. A study has revealed that BMSC-derived exosomal miRNAs in hypoxic conditions facilitate metastatic behaviors of lung cancer cells^8^. It is displayed that major vault protein-mediated exosomal sorting of miR-193a advances colon cancer progression^12^. Also, it is revealed that miR-193a is a repressive miRNA and miR-193a upregulation restrains proliferation and advances apoptosis by regulating oncogenes^13^. Another study has discussed that miR-193a suppresses cell proliferation and invasion while boosts apoptosis in NSCLC^14^. Also, a study has presented that miR-193a gene demethylation attenuates the proliferation of NSCLC cells and advances cell apoptosis^15^. The leucine-rich repeats (LRRs) are 20–29 residue sequence motifs which exist in a variety of proteins with multiple functions^16^. Leucine-rich repeat-containing protein 1 (LRRC1) is a 524 amino acid protein, belonging to cl15309 and pfam13855 family, which is also called LANO (LAP family, without PDZ domain)^17^. According to Almeida et al., expression of LANO/LRRC1 in the inhibition of SCRIB tumor is related to the marker of stem cell in normal and tumor breast epithelial cells^18^. Collectively, it is hypothesized that miR-193a might act as a novel biomarker in NSCLC. For verifying this, we aim to clarify the impacts of miR-193a in NSCLC DDP-resistance via modulating LRRC1 expression.

## Materials and methods

### Ethics statement

The study was consented by the Institutional Review Board of the Affiliated Tumor Hospital of Zhengzhou University, He’nan Cancer Hospital. Ethical agreements were obtained from the donors by written informed consent. All animal experiments were tally with the Guide for the Care and Use of Laboratory Animal by International Committees.

### Study subjects

From July 2015 to July 2018, endoscopic biopsies samples of NSCLC confirmed by pathology in the Affiliated Tumor Hospital of Zhengzhou University, He’nan Cancer Hospital were collected, including 63 NSCLC tumor tissue samples and 63 adjacent normal tissue samples. There were 39 males and 24 females, aged 35–79 years. In terms of tumor-node-metastasis (TNM) stage, 38 cases were of stage I/II and 25 cases of stage III/IV; 35 cases of smoking history and 28 cases of no smoking history; 40 cases of high and middle differentiation and 23 cases of poor differentiation; and the size of the lesions was divided into 40 cases (≥5 cm) and 23 cases (<5 cm). The included patients were those who were pathologically diagnosed as NSCLC^19^, treated with DDP but did not receive radiotherapy or chemotherapy. The excluded patients were those with metastatic NSCLC, long-term use of glucocorticoids, and systemic infectious diseases and other diseases that may affect the study. The patients were separated into DDP-sensitive group (*n* = 34) and DDP-resistant group (*n* = 29) according to computed tomography and response evaluation criteria in solid tumors^20^.

### Cell culture

BMSCs (Shanghai Yaji Biotechnology Co., Ltd., Shanghai, China), human normal bronchial epithelial cells (BEAS-2B), NSCLC cells (H358 and SPC-A-1, the Cell Bank of Type Culture Collection of Chinese Academy of Sciences, Shanghai, China), NSCLC cell A549 (BFN213950, short tandem repeat (STR) report: Amelogenin: X, Y; CSF1PO: 10, 12; D13S317: 11; D16S539: 11, 12; D18S51: 14, 17; D21S11: 29; D3S1358: 16; D5S818: 11; D7S820: 8, 11; D8S1179: 13, 14; FGA: 23; PentaD: 9; PentaE: 7,11; TH01: 8, 9.3; TPOX: 8, 11; vWA: 14), H1299 (BFN213806, STR report: Amelogenin: X; CSF1PO: 12; D13S317: 12; D16S539: 12, 13; D18S51: 16; D19S433: 14; D21S11: 32.2; D2S1338: 23, 24; D3S1358: 17; D5S818: 11; D7S820: 10; D8S1179: 10, 13; FGA: 20; TH01: 6, 9.3; TPOX: 8; vWA: 16, 17, 18) (Shanghai Qingqi Biotechnology Co., Ltd., Shanghai, China), were cultured in 10% fetal bovine serum (FBS)-Roswell Park Memorial Institute (RPMI) 1640 medium (Invitrogen, Carlsbad, CA, USA) under standard conditions (37 °C, 20% O_2_, and 5% CO_2_). A549 and H1299 cell lines resistant to DDP (A549/DDP and H1299/DDP) were induced by continuous exposure and gradually increasing DDP concentration. A549 and H1299 cells in the logarithmic growth phase were initially treated with 0.5 μg/mL DDP (Sigma-Aldrich, St. Louis, MO, USA). When the cells were resistant to the concentration, the DDP concentration gradually increased to 8 μg/mL. When the induced cells survived in 8 μg/mL of DDP for about 2 months and their morphology and activity were normal, cells were confirmed to be DDP-resistant^21^. A549/DDP and H1299/DDP cells were cultured in 10% FBS-RPMI 1640 medium and 2 µg/mL DDP to maintain resistance. The cells in the logarithmic growth phase were utilized for subsequent experiments.

### Identification of BMSCs

Identification of BMSCs immunophenotype: BMSCs of passage 3 were cultured to 80% confluence and detached. Rinsed with 1× phosphate buffered saline (PBS), the cells were counted, centrifuged (2 × 10^5^ cells/tube) and suspended in 1% bovine serum albumin (prepared by 1 × PBS). The cell suspension (20 μL) was added into PE or fluorescein isothiocyanate (FITC)-labeled monoclonal antibody (CD34, CD44, CD73, and CD90, BD company, USA) and reacted in the dark for 30–60 min (A NC group was set). Rinsed three times with 1 × PBS, the cells were suspended in 450 μL 1 × PBS and detected by flow cytometry.

Identification of BMSCs adipogenic induction: when reached 80–90% confluence, BMSCs at P3 were detached by 0.25% trypsin and reckoned. The cells were seeded in the 48-well plate with 7 × 10^3^ cells/well. Next, BMSCs were placed in α-minimum essential medium (α-MEM) containing 1 μM dexamethasone, 10 μg/mL insulin, 500 μM 3-isobutyl-1-methylxanthine and 10% FBS. The liquids in the 48-well plate were changed in half every 2–3 d. After 14 d of induction, oil red O staining was carried out. The cells were cleaned once with 200 μL PBS, fastened with 300 μL 4% neutral formaldehyde solution for 30 min and stained with 300 μL oil red O dye working solution avoiding light for 15 min. Finally, the cells were added with 200 μL PBS and observed for the morphology of lipid droplets under an inverted microscope.

Identification of BMSCs osteogenic induction: BMSCs at passage 3 in a good growth condition with 80–90% confluence were taken out. Two kinds of cells were harvested by trypsinization. The cells suspension was seeded in the 48-well plate with 7 × 10^3^ cells/well. The cells were incubated in α-MEM containing 10^−7^ M dexamethasone, 0.05 mM vitamin C phosphate, 10 mΜ β-glycerophosphate, and 10% FBS with the medium renewed in half every 2–3 d. After 14 d of induction, alkaline phosphatase (ALP) staining was carried out. The cells were cleaned once with 200 μL PBS, fixed with 200 μL fixative solution for 30 s, added with 200 μL staining solution and dyed for 30 min, finally added with 200 μL PBS. The expression of ALP in each group was surveyed under an inverted microscope.

### BMSC-exosome (BMSC-Exo) extraction, identification, and uptake

BMSC-Exo were abstracted by ultra-high speed centrifugation^22^: BMSCs at P4-P6 were harvested. The supernatant was gathered when changing medium. Cells were centrifuged at 4 °C, 500 *g* × 10 min and 12,000 × *g* × 20 min in turn. Using a 0.22-μm well filter, the supernatant was centrifuged at ultra-high speed (100,000 × *g*) × 2 h. The cells were re-suspended in PBS, centrifuged again for 2 h at ultra-high speed, and stored at −80 °C after being re-suspended by PBS.

Exosomes identification: (1) The separated BMSC-Exo were diluted with PBS, and 10 μL sample was absorbed to a copper mesh, then the solution on the surface of the copper mesh was absorbed with filter paper after placed for 5 min. The copper mesh was cleaned once and dyed for 45 s with 2% 10 μL uranyl acetate. The image was developed with 80–120 kv projection electron microscope. The suitable visual field was selected in accordance with the morphology and size of exosomes. (2) The surface antigens (CD63 and CD81) of BMSC-Exo were verified by western blot analysis.

The uptake of A549 and H1299 cells by exosomes was observed by PKH26 staining: a cell coverslip was put to the 24-well plate in advance. A549 and H1299 cell suspensions were seeded in a 24-well plate with 5 × 10^4^ cells/well. After the cell adhered to the wall, the pre-dyed exosomes suspension was appended to the well plate according to the protein concentration of 80 μg/mL and co-cultured for 24 h. The cell coverslip was fastened in 4% paraformaldehyde for 20 min in the dark, dyed with 4′-6-diamidino-2-phenylindole, and blocked with anti-fluorescent quenching agent. The pictures were obtained under a fluorescence microscope.

### miR-193a and LRRC1 modify BMSCs, A549, and A549/DDP cells

A day before BMSCs transfection, cells were separated to the 6-well plate to reach 60% confluence. mimic-negative control (NC), miR-193a mimic, si-NC, si-LRRC1, miR-193a inhibitor + si-NC, or miR-193a inhibitor + si-LRRC1 (all from Shanghai GenePharma Co. Ltd., Shanghai, China) were transfected with BMSCs in strictly accordance with the instructions of lipofectamine 2000 reagent (11668-027, Invitrogen, Carlsbad, CA, USA).

The trypsinized A549 cells and A549/DDP cells were seeded in 6-well plates with 2.0 × 10^6^ cells/well and transfected with mimic NC, miR-193a mimic, si-NC, si-LRRC1, and miR-193a mimic + overexpressed (Oe)-LRRC1 (all from GenePharma) in strictly accordance with the instructions of lipofectamine 2000 reagent.

### MiR-193a and LRRC1 shuttle experiment

BMSC-Exo transfected with mimic NC, miR-193a mimic, si-NC, si-LRRC1, miR-193a inhibitor + si-NC, or miR-193a inhibitor + si-LRRC1 were extracted by ultra-high speed centrifugation and named as EXO-mimic NC, EXO-miR-193a mimic, EXO-si-NC, EXO-si-LRRC1, EXO-miR-193a inhibitor + si-NC, and EXO-miR-193a inhibitor + si-LRRC1.

In order to investigate the effects of BMSC-Exo on the biological functions of NSCLC and DDP-resistant NSCLC cells, A549, A549/DDP, H1299, and H1299/DDP cells were added with 200 μg BMSC-Exo (BMSC-Exo group) or PBS (control group). Forty-eight hours later, cells were harvested for the following experiments.

In order to investigate the effects of miR-193a in exosomes on the biological functions of A549, H1299, A549/DDP, and H1299/DDP cells, these cells were assigned into six groups and treated with 200 μg BMSC-Exo transfected with miR-193a mimic NC, 200 μg BMSC-Exo transfected with miR-193a mimic, 200 μg BMSC-Exo transfected with si-LRRC1 plasmid NC, 200 μg BMSC-Exo transfected with si-LRRC1 plasmid, 200 μg BMSC-Exo transfected with miR-193a inhibitor and si-LRRC1 plasmid NC or 200 μg BMSC-Exo transfected with miR-193a inhibitor and si-LRRC1 plasmid. Forty-eight hours after treatment, cells were gathered for the following experiments.

### RT-qPCR

The total RNA in tissues, cells and exosomes was extracted and determined on the basis of Trizol reagent (Invitrogen). The RNA was reverse-transcribed into complementary DNA in the light of the reverse transcription kit (K1621, Fermentas, Maryland, NY, USA). Gene expression was verified by fluorescence quantitative PCR Kit (Takara, Dalian, China). A real-time fluorescence quantitative PCR instrument (Thermo Fisher Scientific, Massachusetts, USA) was adopted for detection. U6 was the loading control of miR-193a while glyceraldehyde phosphate dehydrogenase (GAPDH) of LRRC1, P-glycoprotein (P-gp), topoisomerase II alpha (TopoIIα) and glutathione s-transferases pi (GST-π). 2^−△△Ct^ method was utilized for data assessment^23^. The primer sequences of miR-193a, LRRC1, P-gp, TopoIIα, and GST-π were devised and compounded by Shanghai Qi Yin Biotechnology Co. Ltd. (Shanghai, China) (Table 1).

**Table 1:** Primer sequence.

Gene	Sequence
miR-193a	F: 5′-AATTTGGGTCTTTGCGGGCGAGATGAT-3′
	R: 5′-CTAGATCATCTCGCCCGCAAAGACCCA-3′
LRRC1	F: 5′-TCCTTACCAAAAGAGATCGG-3′
	R: 5′-GGTAGATGCAGCAACCTGT-3′
P-gp	F: 5′-AGCAGAGGATCGCCATTGC-3′
	R: 5′-CTGAACCACTGCTTCGCTTTC-3′
TopoIIα	F: 5′-GAAACGGAATCCTTGGTCAGAT-3′
	R: 5′-TTTCGGCTGCTGCTCTCCTA-3′
GST-π	F: 5′-CAGGGAGGCAAGACCTTCATT-3′
	R: 5′-GGGCTAGGACCTCATGGATCA-3′
U6	F: 5′-CTCGCTTCGGCAGCACA-3′
	R: 5′-AACGCTTCACGAATTTGCGT-3′
GAPDH	F: 5′-TCCCATCACCATCTTCCA-3′
	R: 5′-CATCACGCCACAGTTTTCC-3′

*F* forward, *R* reverse, *miR-193a* microRNA-193a, *P-gp* P-glycoprotein, *TopoIIα* topoisomerase II alpha, *GST-π* glutathione s-transferases pi, *GAPDH* glyceraldehyde phosphate dehydrogenase.

### Western blot analysis

Total proteins in tissues, cells and exosomes were extracted by radio-immunoprecipitation assay lysis buffer (R0010, Solarbio Science & Technology Co. (Beijing, China). The protein concentration was determined by bicinchoninic acid kit (Shanghai Yanxi Biotechnology Co., Ltd., Shanghai, China). The abstracted protein was appended to the loading buffer, boiled at 95 °C for 10 min (30 μg/well), and isolated with 10% sodium dodecyl sulfate polyacrylamide gel electropheresis. The protein was transferred to a polyvinylidene fluoride membrane by a semidry electrophoretic transfer apparatus (Sigma-Aldrich, SF, CA, USA) and sealed with 5% bovine serum albumin (AmyJet Scientific Inc., Wuhan, Hubei, China). The primary antibody LRRC1 (1:500), P-gp (1:500), TopoIIα (1:10000), GST-π (1:1000, Abcam, Cambridge, MA, USA), CD63 (1:100, BD Biosciences, Lake Franklin, New Jersey, USA), and CD81 (1:200, Santa Cruz Biotechnology, Santa Cruz, CA, USA) were appended. The horseradish peroxide-conjugated secondary antibody (1:1000, AmyJet Scientific Inc., Wuhan, China) was incubated for 1 h. The image was developed by chemiluminescence reagent. GADPH (1:10,000, Abcam) was utilized as a loading control. Bio-rad Gel Doc EZ imager (Bio-Rad, California, USA) was utilized to development while Image J software (National Institutes of Health, Bethesda, MD, USA) to protein band evaluation.

### Colony formation assay

Cells were detached and centrifuged to obtain the cell precipitation. The cell precipitation was re-suspended, enumerated, adjusted to 1 × 10^5^ cell/mL and diluted to 1 × 10^3^ cells/mL. An appropriate amount of cell suspension was seeded in the 6-well plate, which was supplemented with culture medium to 4 mL. The cells were uniformly dispersed and incubated in a 5% CO_2_ incubator for 2–3 weeks. The cell culture was terminated and the culture medium was discarded when colonies could be seen by naked eyes. The cells were fastened by methanol for 15 min and dyed with crystal violet staining solution for 10 min. The colony number visible to naked eyes was counted, and the colony rate = (colony number/seeded cell number) × 100%.

### Cell counting kit (CCK)-8 assay

The cells were detached and centrifuged to obtain the cell precipitation. The cell precipitation was re-suspended and counted. The cell suspension was diluted and adjusted to 1 × 10^4^ cells/mL. Followed by that, the diluted cell suspension (200 μL) was absorbed and appended to a 96-well plate. The experiment was completed by following the instructions of CCK-8 (Dojindo, Tokyo, Japan). Adherent cells were treated with DDP of gradient concentration (0, 1, 5, 10, 15, 20, 40, 50, 60, 70, 80 μg/mL). After cultured for 48 h, the culture medium was replaced with 10% CCK-8 fresh medium, and the cells were incubated for 3 h at 5% CO_2_. Absorbance (A) values were detected at 450 nm. The inhibitory rate of DDP on the growth of A549 cells, A549/DDP cells, H1299 cells and H1299/DDP cells were enumerated, respectively. The growth inhibition rate = (1 − A value in the experimental group/A value in the control group) × 100%. The inhibition curve was plotted with the concentration of DDP as the abscissa and the growth inhibition rate as the ordinate. Half inhibitory concentration (IC50) value and the resistance index were reckoned. miR-193a and LRRC1 expression in A549/DDP and H1299/DDP cells treated with different concentrations of DDP was measured.

Detached by 0.25% trypsin and prepared into single cell suspension, the cells were suspended with a small amount of culture medium and reckoned. The cell suspension was diluted and adjusted to 1 × 10^4^ cells/mL. The diluted cell suspension (200 μL) was absorbed and appended to the 96-well plate. According to CCK-8 (Dojindon) specifications, the cells were cultured for 24, 48, and 72 h, and incubated with 10 μL CCK-8 solution for 3 h. A values (450 nm) were detected by a microplate reader.

### Flow cytometry

Seeded in the 6-well plate with 2 × 10^6^ cells/well and detached with 0.25% trypsin (excluding ethylene diamine tetraacetic acid), the cells were centrifuged to remove the supernatant. According to the instructions of Annexin V-FITC cell apoptosis detection kit (K201-100, Biovision, USA), cells were added with 500 μL loading buffer, 5 μL Annexin V-FITC and 10 μL propidium iodide (PI) solution and reacted for 15–20 min. The apoptosis rate was tested by flow cytometry (BD Biosciences).

### Transwell assay

A 8-μm well size Transwell chamber (Corning, N.Y., USA) was adopted for this experiment. The upper surface of Transwell chamber bottom membrane was covered with 100 μL of 50 mg/L Matrigel (1:40). Cell suspension (100 μL, 2 × 10^5^ cells) was appended to the upper chamber, and 600 µL RPMI 1640 medium containing 20% FBS was supplemented to the lower chamber. Incubated for 24 h, the cells were fixed with the methanol for 10 min and dyed with 1% crystal violet staining solution. Eight fields of view were randomly selected under the microscope and the cell number was counted. The cell migration experiment did not pre-coat the matrigel, and the rest of the steps were same as the invasion experiment. Three parallel wells were set for each group.

### Dual luciferase reporter gene assay

The targeting relationship between miR-193a and LRRC1 and the binding site between miR-193a and LRRC1 3′-untranslated region (3′UTR) were forecasted by bioinformatics software http://starbase.sysu.edu.cn/. LRRC1 3′UTR promoter region containing miR-193a binding site was composed and the wild-type (WT) plasmid of LRRC1 3′UTR (LRRC1-WT) was constructed. Based on this plasmid, a site mutation kit (Takara) was used to mutate the miR-193a binding site on LRRC1-WT to construct an LRRC1 3′UTR mutant (MUT) plasmid (LRRC1-MUT). A549 and H1299 cells were seeded into the 96-well plate and cultured to 70% confluence. Correctly sequenced LRRC1-WT or LRRC1-MUT plasmids with mimics NC or miR-193a mimics were co-transfected to A549 and H1299 cells by Lipofectamine 2000 reagent. After 48-h transfection, cells were lysed and the luciferase activity was verified by luciferase detection kit.

### RNA pull-down assay

Biotin-labeled RNA (3 μg), Bio‐NC‐probe, and Bio‐LRRC1‐probe were incubated with RNA structure buffer (100 μL). A549 and H1299 cells (10^7^ cells) were added with 1 mL Trizol reagent (Life Technologies, MA, USA) to extract total RNA. Then, total RNA (1 mg) was mingled with biotin‐labeled RNA and incubated with pre-washed streptavidin beads for 1 h to separate beads before centrifugation (1006.2 × *g*, 4 °C, 3 min). The beads were rinsed to remove non‐specific binding. The hybridized RNA remained was reversely transcribed and quantified by SuperScript III First‐Strand Synthesis System kit (Invitrogen) and Maxima SYBR Green qPCR Master Mix (2X) kit (Thermo Fisher Scientific) with GAPDH as an internal control.

### Tumor xenograft in nude mice

BALB/c nude mice (*n* = 60, Experimental Animal Center of Zhengzhou University, Zhengzhou, China), aged at 4–6 weeks were fed in a specific pathogen-free grade environment with a constant temperature of 24–27 °C and a humidity of 45–50%.

In order to verify the effect of miR-193a on A549 cells and A549/DDP cells in vivo, we divided the nude mice into 2 groups: mimic-NC group and miR-193a mimic group. Each nude mouse was subcutaneously injected with the transfected single cell suspension (4 × 10^6^ cells/mL) into the back.

In order to verify the role of BMSC-Exo containing miR-193a in suppressing NSCLC or improving NSCLC DDP sensitivity in vivo, we divided the nude mice into Control, BMSC-Exo, EXOmimic-NC, EXOmiR-193a mimic groups. When the mean tumor volume reached 100 mm^3^, the extracted exosomes were intratumorally injected into mice at a dose of 200 μg per mouse every 2 d for 10 times. After 4 weeks, the tumor was removed, the length and width of the tumor were measured with a ruler, and the weight of the tumor was weighed with an electronic balance. Tumor volume = (length × width^2^)/2.

### Statistical analysis

All data were interpreted by SPSS 19.0 software (IBM Corp., Armonk, NY, USA). The measurement data were represented as mean ± standard deviation. The differences between groups were conducted by *t*-test, while comparisons among multiple groups were assessed by one-way analysis of variance (ANOVA), and Tukey’s multiple comparisons test was used after ANOVA. Pearson was adopted for correlation analysis. Fisher test was utilized to verify the relationship between miR-193a expression and clinicopathological features of NSCLC patients. Statistical significance was set at *P* < 0.05.

## Results

### MiR-193a expression decreases and LRRC1 expression increases in DDP-resistant NSCLC tissues; miR-193a expression is related to TNM stage and differentiation degree of NSCLC patients

**Fig. 1:**
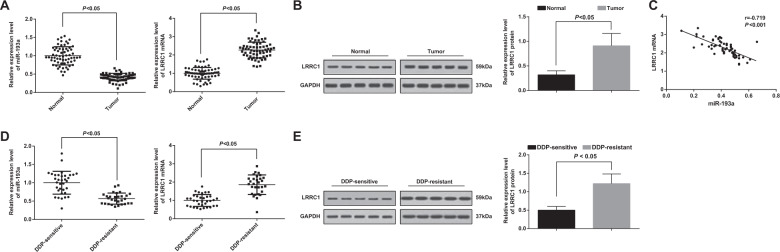
MiR-193a expression is decreased and LRRC1 expression is increased in DDP-resistant NSCLC tissues. **a** miR-193a and LRRC1 mRNA expression in NSCLC tumor tissues and adjacent normal tissues. **b** LRRC1 protein expression in NSCLC tumor and adjacent normal tissues. **c** The correlation analysis between LRRRC1 mRNA expression and miR-193a expression in NSCLC. **d** miR-193a and LRRC1 mRNA expression in the DDP-sensitive group and the DDP-resistant group. **e** LRRC1 protein expression in the DDP-sensitive group and the DDP-resistant group. Measurement data were depicted as mean ± standard deviation, comparisons between two groups were conducted by *t-*test.

miR-193a and LRRC1 expression in NSCLC tumor tissues and adjacent normal tissues, and in DDP-resistant tissues and DDP-sensitive tissues were tested by western blot analysis and RT-qPCR. The results manifested that miR-193a expression in NSCLC tumor tissues decreased while LRRC1 expression elevated relative to that in the adjacent normal tissues (both *P* < 0.05) (Fig. 1a, b). As depicted in Fig. 1c, a negative relation was presented between miR-193a and LRRC1 mRNA expression in NSCLC (*r* = −0.719, *P* < 0.001). Moreover, miR-193a expression declined while LRRC1 expression increased in DDP-resistant tissues relative to those in DDP-sensitive tissues (both *P* < 0.05) (Fig. 1d, e).

NSCLC patients were distributed into the low expression group and the high expression group in the light of the median miR-193 expression. The connection between miR-193a expression and clinicopathological traits of NSCLC patients was analyzed. The results demonstrated that TNM stage and differentiation degree of NSCLC patients were connected with miR-193a expression (both *P* < 0.05), whereas age, gender, history of smoking and size of the lesions were not associated with miR-193a expression (all *P* > 0.05) (Table 2).

**Table 2:** Relationship between miR-193a expression and clinicopathological features of NSCLC patients.

Characteristics	*n*	miR-193a expression		*P*
		Low expression (*n* = 32)	High expression (*n* = 31)	
Age (year)				
<60	36	19	17	0.801
≥60	27	13	14	
Gender				
Male	39	18	21	0.439
Female	24	14	10	
TNM stage				
I–II	38	9	29	<0.001
III–IV	25	23	2	
Smoking history				
Smoking	35	20	15	0.315
Non-smoking	28	12	16	
Differentiation degree				
High and middle differentiation	40	16	24	0.036
Poor differentiation	23	16	7	
Size of lesions (cm)				
≥5	40	23	17	0.196
<5	23	9	14	

*miR-193a* microRNA-193a; *TNM stage* tumor-node-metastasis stage.

The data in this table was enumeration data, and verified by chi-square test.

### MiR-193a expression is reduced and LRRC1 expression is raised in NSCLC DDP-resistant cells; and LRRC1 is a target gene of miR-193a

**Fig. 2:**
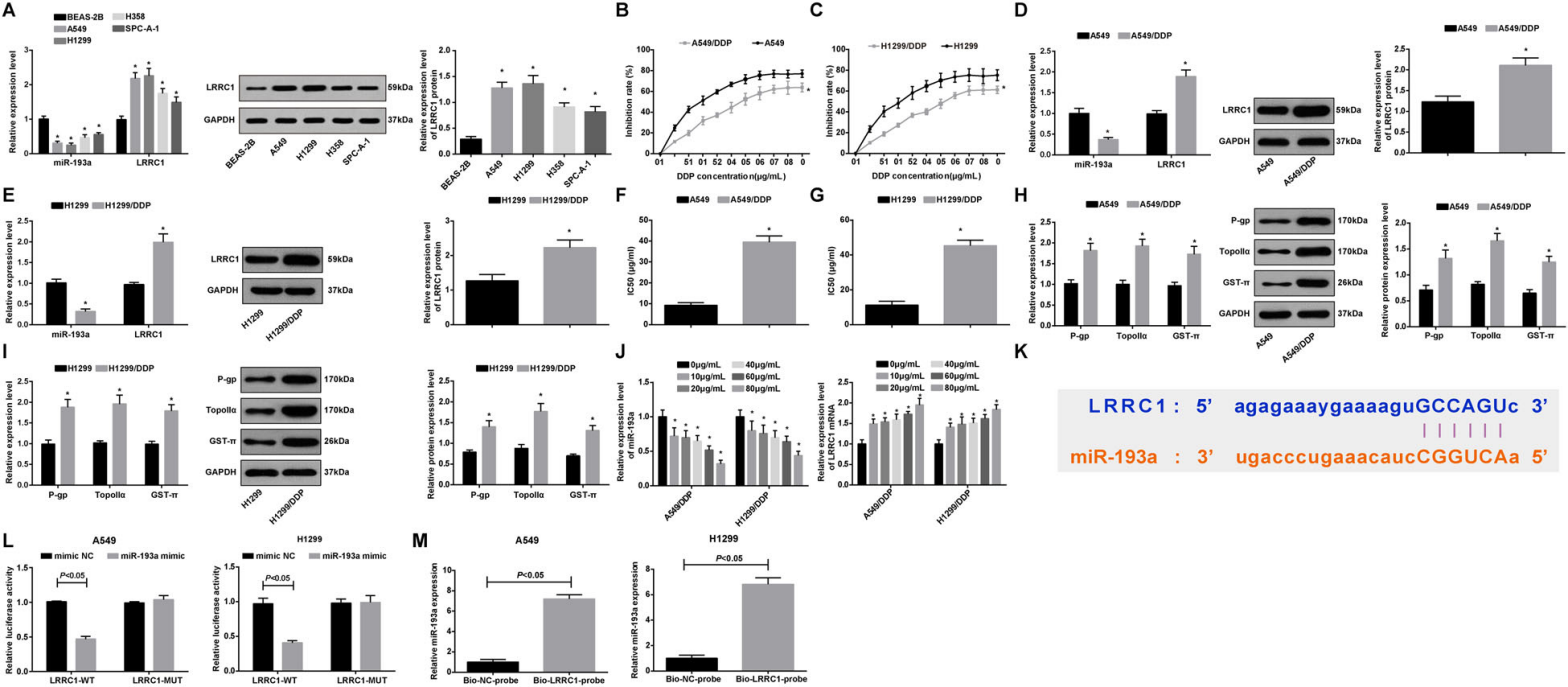
MiR-193a expression is reduced and LRRC1 expression is elevated in NSCLC DDP cells; and LRRC1 is a target gene of miR-193a. **a** Expression of miR-193a and LRRC1 in BEAS-2B, A549, H1299, H358, and SPC-A-1 cells. **b** Comparison of proliferation inhibition rate of A549 cells and A549/DDP cells at different concentrations of DDP. **c** Comparison of proliferation inhibition rate of H1299 cells and H1299/DDP cells at different concentrations of DDP. **d** Expression of miR-193a and LRRC1 in A549 and A549/DDP cells. **e** Expression of miR-193a and LRRC1 in H1299 cells and H1299/DDP cells. **f** Comparison of IC50 values between A549 cells and A549/DDP cells. **g** Comparison of IC50 values between H1299 cells and H1299/DDP cells. **h** Expression of P-gp, TopoII, and GST-π in A549 and A549/DDP cells. **i** Expression of P-gp, TopoII, and GST-π in H1299 cells and H1299/DDP cells. **j** Expression of miR-193a and LRRC1 in A549/DDP and H1299/DDP cells under different concentrations of DDP. **k** The target relationship between miR-193a and LRRC1 predicated by Starbase website. **l** The target relationship between miR-193a and LRRC1 verified by dual luciferase reporter gene assay. **m** The regulatory relationship between LRRC1 and miR-193a in A549 and H1299 cells verified by RNA pull-down assay. *P < 0.05 vs. BEAS-2B cells (**a**). *P < 0.05 vs. A549 cells (**b**, **d**, **f**, **h**). *P < 0.05 vs. H1299 cells (**c**, **e**, **g**, **i**). *P < 0.05 vs. 0 μg/mL DDP treatment (**j**). Measurement data were depicted as mean ± standard deviation, comparisons between two groups were assessed by *t-*test while those among multiple groups were assessed by one-way ANOVA followed with Tukey’s multiple comparisons test, the experiment was repeated three times.

Western blot analysis and RT-qPCR were utilized to detect miR-193a and LRRC1 expression in BEAS-2B, A549, H1299, H358, and SPC-A-1 cells. Two NSCLC cell lines with relative low miR-193a expression and high LRRC1 expression were screened out. The results indicated that in contrast with BEAS-2B cell line, miR-193a expression was reduced and LRRC1 expression was elevated in A549, H1299, H358, and SPC-A-1 cell lines (all *P* < 0.05). miR-193a expression in A549 and H1299 cell lines was relatively lower, and LRRC1 expression was relatively higher (all *P* < 0.05). Therefore, A549 and H1299 cells were chosen for subsequent cell experiments (Fig. 2a).

CCK-8 assay was used to test the growth and IC50 of A549, A549/DDP, H1299, and H1299/DDP cells. It was presented that the growth inhibition rate of A549, A549/DDP, H1299, and H1299/DDP cells enhanced with the increase of DDP concentration, showing a dose-dependent manner, and the growth inhibition rates of A549/DDP and H1299/DDP cells were remarkably lower than that of A549 and H1299 cells (both *P* < 0.05) (Fig. 2b, c).

miR-193a and LRRC1 expression in cells were verified by western blot analysis and RT-qPCR. It was displayed that miR-193a expression in A549/DDP and H1299/DDP cells was dramatically lower while LRRC1 expression was higher than those in A549 and H1299 cells (all *P* < 0.05) (Fig. 2d, e).

According to the linear regression equation, IC50 of A549/DDP and H1299/DDP cells were higher than that of A549 cells and H1299 cells (both *P* < 0.05). The drug resistance indices of A549/DDP and H1299/DDP cells to DDP were 39.53 and 45.35, respectively, indicating that the resistant cells could be used in subsequent cell experiments (Fig. 2f, g). RT-qPCR and western blot analysis verified that P-gp, TopoII and GST-π expression in A549/DDP and H1299/DDP cells elevated relative to those of A549 and H1299 cells (all *P* < 0.05) (Fig. 2h, i). RT-qPCR revealed that miR-193a was decreased while LRRC1 was increased in A549/DDP and H1299/DDP cells with the increase of the concentration of DDP (*P* < 0.05) (Fig. 2j).

miR-193a could target LRRC1 which had predicted by starbase website (Fig. 2k). Dual luciferase reporter gene assay (Fig. 2I) suggested that miR-193a mimic impaired the luciferase activity of LRRC1-WT (both *P* < 0.05), while had no effects on that of LRRC1-MUT (*P* > 0.05). RNA pull-down assay (Fig. 2m) revealed that LRRC1 could bind to miR-193a, indicating that LRRC1 was directly targeted by miR-193a.

### miR-193a upregulation and LRRC1 downregulation inhibit NSCLC cell progression and mouse tumor growth in NSCLC

**Fig. 3:**
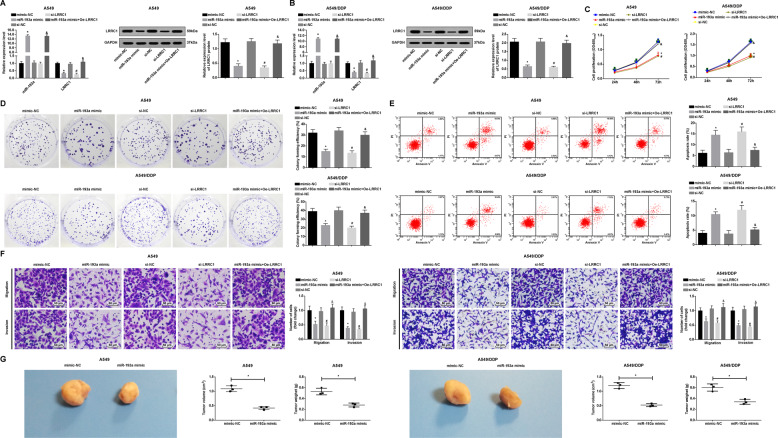
miR-193a upregulation and LRRC1 downregulation inhibit NSCLC cell progression and mouse tumor growth in NSCLC. **a** Effect of upregulation of miR-193a and downregulation of LRRC1 on the expression of miR-193a and LRRC1 in A549 cells. **b** Effect of upregulation of miR-193a and downregulation of LRRC1 on the expression of miR-193a and LRRC1 in A549/DDP cells. **c** Effects of upregulation of miR-193a and downregulation of LRRC1 on proliferation ability of A549 and A549/DDP cells. **d** Effects of upregulation of miR-193a and downregulation of LRRC1 on colony formation ability of A549 and A549/DDP cells. **e** Effects of upregulation of miR-193a and downregulation of LRRC1 on apoptosis of A549 and A549/DDP cells. **f** Effects of upregulation of miR-193a and downregulation of LRRC1 on invasion and migration abilities of A549 and A549/DDP cells. **g** Effect of upregulation of miR-193a on tumorigenesis of A549 and A549/DDP cells in nude mice. *P < 0.05 vs. the mimic-NC group. ^#^*P* < 0.05 vs. the si-NC group. ^&^*P* < 0.05 vs. the miR-193a mimic group. Measurement data were depicted as mean ± standard deviation, comparisons between two groups were assessed by *t-*test while those among multiple by one-way ANOVA followed with Tukey’s multiple comparisons test, the experiment was repeated three times.

RT-qPCR and western blot analysis were utilized to detect the effect of upregulated miR-193a and downregulated LRRC1 on target gene expression in NSCLC cells. The results showed that miR-193a overexpression elevated miR-193a and decreased LRRC1 expression in A549 cells and A549/DDP cells. LRRC1 downregulation reduced LRRC1 expression in A549 cells and A549/DDP cells. LRRC1 upregulation reversed miR-193 overexpression-induced effects on LRRC1 expression. No difference was witnessed in LRRC1 expression in A549 cells and A549/DDP cells treated with mimic NC, si-NC, or miR-193a mimic + Oe-LRRC1 (Fig. 3a, b).

Colony formation, CCK-8 and Transwell assays reported that miR-193a upregulation or LRRC1 downregulation suppressed cell proliferation, invasion, and migration of A549 cells and A549/DDP cells. LRRC1 restoration followed by miR-193a upregulation enhanced cell proliferation, invasion, and migration of A549 cells and A549/DDP cells. There was no marked difference of cell proliferation, invasion, and migration in cell treated with mimic NC, si-NC, or miR-193a mimic + Oe-LRRC1 (Fig. 3c, d, f).

Annexin V-FITC/PI double staining indicated that miR-193a elevation or LRRC1 reduction raised apoptosis rate of A549 and A549/DDP cells. LRRC1 overexpression negated the promoting effect of miR-193a upregulation on apoptosis rate of A549 and A549/DDP cells. There was no marked difference of apoptosis rate in cell treated with mimic NC, si-NC, or miR-193a mimic + Oe-LRRC1 (Fig. 3e).

The effect of upregulated miR-193a on NSCLC cells in vivo was tested. It was revealed that miR-193a upregulation retarded tumor volume and weight increments after 4 weeks (Fig. 3g).

### Identification of BMSCs and exosomes; exosomes uptake test

The surface antigens of BMSCs were tested by flow cytometry. The results showed that in BMSCs of passage 3, CD44, CD73, and CD90 were positively expressed, while CD34 was negatively expressed (Supplementary Fig. 1A).

BMSCs adipogenic induction indicated that after adipogenic differentiation of BMSCs for about 2 weeks, it was seen that lipid droplets were formed in cytoplasm, a large number of oil droplet vacuoles could be seen after oil red O staining, and lipid droplets presented rosette structure (Supplementary Fig. 1B). The results of BMSCs osteogenic induction reported that a large number of brown calcium nodules were observed by ALP staining under the inverted microscope after 14 d of osteogenic induction. After staining with ALP dye, the red cytoplasm was shown and the formation of calcified nodules could be seen under the microscope (Supplementary Fig. 1C).

The morphology of BMSC-Exo was surveyed by a transmission electron microscope (TEM). The results indicated that the small vesicles in circular or oval shape were distributed in the visual field under the TEM, which were in heterogeneity, and the diameter was 30–100 nm. In addition, the membranous structure of the vesicles was observed in the outer periphery of the vesicle, in which low-density substance was included (Supplementary Fig. 1D). Western blot analysis reported that CD63 and CD81 expression in BMSC-Exo were at a high level (Supplementary Fig. 1E). The results of uptake experiment displayed that BMSC-Exo were labeled by PKH26 dye and co-cultured with A549 and H1299 cells, and a large number of BMSC-Exo labeled with green fluorescence were observed to be ingested by A549 and H1299 cells under a confocal microscope (Supplementary Fig. 1F).

### BMSC-Exo inhibit NSCLC cell progression and mouse tumor growth in NSCLC

**Fig. 4:**
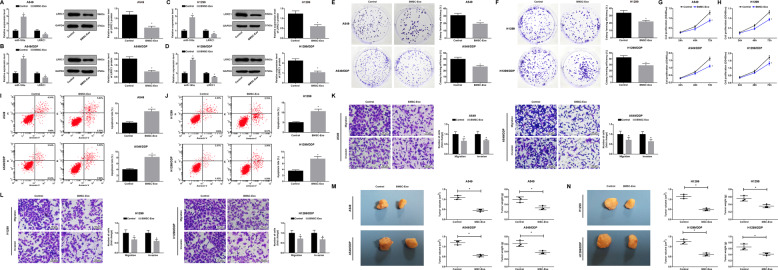
BMSC-Exo inhibit NSCLC cell progression and mouse tumor growth in NSCLC. **a** Effect of BMSC-Exo on the expression of miR-193a and LRRC1 in A549 cells. **b** Effect of BMSC-Exo on the expression of miR-193a and LRRC1 in A549/DDP cells. **c** Effect of BMSC-Exo on the expression of miR-193a and LRRC1 in H1299 cells. **d** Effect of BMSC-Exo on the expression of miR-193a and LRRC1 in H1299/DDP cells. **e** Impacts of BMSC-Exo on colony formation ability of A549 and A549/DDP cells. **f** Impacts of BMSC-Exo on colony formation ability of H1299 and H1299/DDP cells. **g** Impacts of BMSC-Exo on proliferation ability of A549 and A549/DDP cells. **h** Impacts of BMSC-Exo on proliferation ability of H1299 and H1299/DDP cells. **i** Impacts of BMSC-Exo on apoptosis of A549 and A549/DDP cells. **j** Impacts of BMSC-Exo on apoptosis of H1299 and H1299/DDP cells. **k** Impacts of BMSC-Exo on migration and invasion abilities of A549 and A549/DDP cells. **l** Impacts of BMSC-Exo on migration and invasion abilities of H1299 and H1299/DDP cells. **m** Effect of BMSC-Exo injection on A549 and A549/DDP cell tumorigenesis in nude mice. **n** Effect of BMSC-Exo injection on tumorigenesis of H1299 and H1299/DDP cells in nude mice. *P < 0.05 vs. the control group. Measurement data were depicted as mean ± standard deviation, comparisons between two groups were assessed by *t-*test. The experiment was repeated three times.

The impact of BMSC-Exo on target gene expression in NSCLC cells was tested by RT-qPCR and western blot analysis. It was found that BMSC-Exo enhanced miR-193a expression and decreased LRRC1 expression in A549, A549/DDP, H1299, and H1299/DDP cells (Fig. 4a–d). Colony formation, CCK-8 and Transwell assays and Annexin V-FITC/PI double staining reported that BMSC-Exo impaired cell colony-forming, proliferation, invasion and migration abilities while enhanced apoptosis of A549, A549/DDP, H1299, and H1299/DDP cells (Fig. 4e–l).

The effect of BMSC-Exo on NSCLC cells in vivo was tested by tumor xenograft in nude mice, and the result demonstrated that BMSC-Exo reduced tumor volume and weight after 4 weeks (Fig. 4m, n).

### BMSC-Exo with upregulated miR-193a and downregulated LRRC1 suppress colony formation of NSCLC cells

**Fig. 5:**
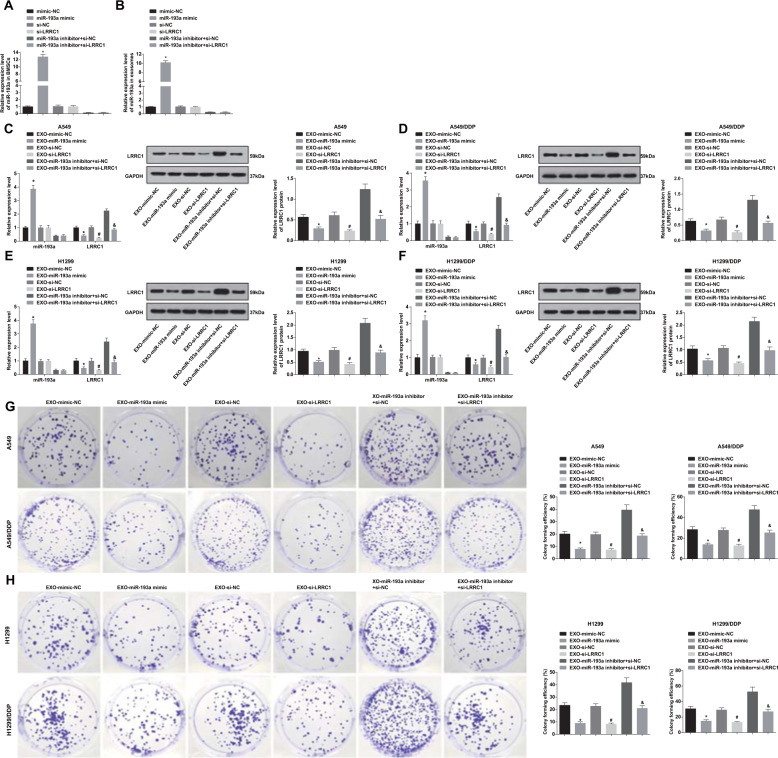
BMSC-Exo with upregulated miR-193a and downregulated LRRC1 suppress colony formation of NSCLC cells. **a** miR-193a expression in BMSCs. **b** miR-193a expression in BMSC-Exo. **c** The effect of BMSC-Exo with upregulated miR-193a and downregulated LRRC1 on miR-193a and LRRC1 expression in A549 cells. **d** The effect of BMSC-Exo with upregulated miR-193a and downregulated LRRC1 on miR-193a and LRRC1 expression in A549/DDP cells. **e** The effect of BMSC-Exo with upregulated miR-193a and downregulated LRRC1 on miR-193a and LRRC1 expression in H1299 cells. **f** The effect of BMSC-Exo with upregulated miR-193a and downregulated LRRC1 on miR-193a and LRRC1 expression in H1299/DDP cells. **g** Impacts of BMSC-Exo with upregulated miR-193a and downregulated LRRC1 on colony formation ability of A549 and A549/DDP cells. **h** Impacts of BMSC-Exo with upregulated miR-193a and downregulated LRRC1 on colony formation ability of H1299 and H1299/DDP cells. In (**a**, **b**), *P < 0.05 vs. mimic-NC group. In (**c**–**h**), *P < 0.05 vs. EXO-mimic-NC group. ^#^*P* < 0.05 vs. EXO-si-NC group. ^&^*P* < 0.05 vs. EXO-miR-193a inhibitor + si-NC group. Measurement data were depicted as mean ± standard deviation, comparisons among multiple groups were assessed by one-way ANOVA followed with Tukey’s multiple comparisons test, the experiment was repeated three times.

RT-qPCR was utilized to test miR-193a expression in BMSCs and exosomes after transfected with miR-193a mimic, inhibitor, or si-LRRC1. The results displayed that (Fig. 5a, b) miR-193a mimic elevated miR-193a expression in BMSCs and exosomes. si-LRRC1 transfection imposed no effect on miR-193a expression in BMSCs and exosomes. Relative to BMSCs and exosomes transfected with miR-193a inhibitor and si-NC, miR-193a expression showed no obvious difference in those transfected with miR-193a inhibitor and si-LRRC1.

RT-qPCR and western blot analysis demonstrated the effects of BMSC-Exo with upregulated miR-193a and downregulated LRRC1 on the target gene expression in NSCLC cells. The results reported that miR-193a-overexpressed BMSC-Exo raised miR-193a expression while reduced LRRC1 expression versus to mimic-NC-transfected BMSC-Exo. LRRC1-downregulated BMSC-Exo imposed no effect on miR-193a expression but decreased LRRC1 expression versus to si-NC-transfected BMSC-Exo. BMSC-Exo transfected with miR-193a inhibitor + si-LRRC1 reduced LRRC1 expression verus to BMSC-Exo transfected with miR-193a inhibitor + si-NC. There was no marked difference of LRRC1 expression in cells treated with BMSC-Exo transfected with mimic NC, si-NC, or miR-193a inhibitor + si-LRRC1 (Fig. 5c–f).

Colony formation assay revealed that cell colony formation was suppressed by miR-193a-overexpressed or LRRC1-downregulated BMSC-Exo versus to mimic-NC- or si-NC-transfected BMSC-Exo. In contrast to cells treated with miR-193a inhibitor + si-NC-transfected BMSC-Exo, colony formation ability was reduced in cells treated with miR-193a inhibitor + si-LRRC1-transfected BMSC-Exo. There was no obvious difference of the colony formation ability in cells treated with mimic NC-, si-NC-, or miR-193a inhibitor + si-LRRC1-transfected BMSC-Exo (Fig. 5g, h).

### BMSC-Exo with upregulated miR-193a and downregulated LRRC1 suppress proliferation and reduces cisplatin resistance of NSCLC cells

**Fig. 6:**
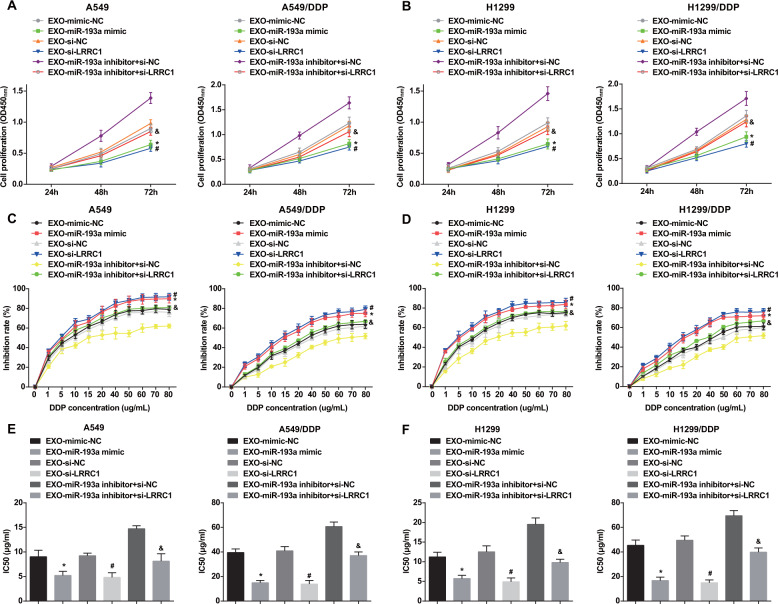
BMSC-Exo with upregulated miR-193a and downregulated LRRC1 suppress proliferation and reduces cisplatin resistance of NSCLC cells. **a** Impacts of BMSC-Exo with upregulated miR-193a and downregulated LRRC1 on proliferation ability of A549 and A549/DDP cells. **b** Impacts of BMSC-Exo with upregulated miR-193a and downregulated LRRC1 on proliferation ability of H1299 and H1299/DDP cells. **c** Impacts of BMSC-Exo with upregulated miR-193a and downregulated LRRC1 on growth inhibition rate of A549 and A549/DDP cells at different concentration of DDP. **d** Impacts of BMSC-Exo with upregulated miR-193a and downregulated LRRC1 on growth inhibition rate of H1299 and H1299/DDP cells at different concentration of DDP. **e** Impacts of BMSC-Exo with upregulated miR-193a and downregulated LRRC1 on IC50 value of A549 and A549/DDP cells. **f** Impacts of BMSC-Exo with upregulated miR-193a and downregulated LRRC1 on IC50 value of H1299 and H1299/DDP cells; *P < 0.05 vs. EXO-mimic-NC group. ^#^*P* < 0.05 vs. EXO-si-NC group. ^&^*P* < 0.05 vs. EXO-miR-193a inhibitor + si-NC group. Measurement data were depicted as mean ± standard deviation, comparisons among multiple groups were assessed by one-way ANOVA followed with Tukey’s multiple comparisons test, the experiment was repeated three times.

CCK-8 assay revealed that cell proliferation was repressed by miR-193a-overexpressed or LRRC1-downregulated BMSC-Exo in relative to mimic-NC- or si-NC-transfected BMSC-Exo. In contrast to cells treated with miR-193a inhibitor + si-NC-transfected BMSC-Exo, cell proliferation was impaired in cells treated with miR-193a inhibitor + si-LRRC1-transfected BMSC-Exo. There was no obvious difference of the proliferation in cells treated with mimic NC-, si-NC-, or miR-193a inhibitor + si-LRRC1-transfected BMSC-Exo (Fig. 6a, b).

Cell viability and IC50 value were also detected by CCK-8 assay. The results showed that the growth inhibition rate of cells was increased with the increase of DDP concentration, showing a dose-dependent manner. The growth inhibition rate of cells treated with miR-193a-overexpressed or LRRC1-downregulated BMSC-Exo, or BMSC-Exo transfected with miR-193a inhibitor + si-LRRC1 was increased and the IC50 value of DDP was reduced with respect to mimic-NC- or si-NC-transfected BMSC-Exo or BMSC-Exo transfected with miR-193a inhibitor + si-NC, respectively. There was no significant difference in the growth inhibition rate and IC50 value of DDP in cells treated with BMSC-Exo transfected with mimic NC, si-NC, or miR-193a inhibitor + si-LRRC1 (Fig. 6c–f).

### BMSC-Exo with upregulated miR-193a and downregulated LRRC1 restrain migration and invasion while promote apoptosis of NSCLC cells, and suppress tumor growth in mice with NSCLC

**Fig. 7:**
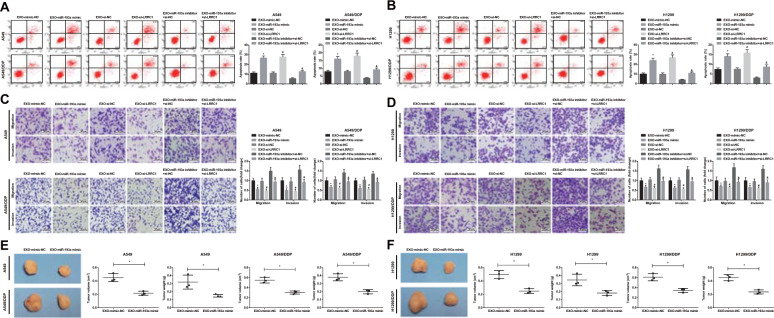
BMSC-Exo with upregulated miR-193a and downregulated LRRC1 restrain migration and invasion while promote apoptosis of NSCLC cells, and suppress tumor growth in mice with NSCLC. **a** Effects of BMSC-Exo with upregulated miR-193a and downregulated LRRC1 on apoptosis of A549 and A549/DDP cells. **b** Effects of BMSC-Exo with upregulated miR-193a and downregulated LRRC1 on apoptosis of H1299 and H1299/DDP cells. **c** Impacts of BMSC-Exo with upregulated miR-193a and downregulated LRRC1 on invasion and migration abilities of A549 and A549/DDP cells. **d** Impacts of BMSC-Exo with upregulated miR-193a and downregulated LRRC1 on invasion and migration abilities of H1299 and H1299/DDP cells. **e** The effect of BMSC-Exo with upregulated miR-193a on tumorigenesis of A549 and A549/DDP cells. **f** The effect of BMSC-Exo with upregulated miR-193a on tumorigenesis of H1299 and H1299/DDP cells. *P < 0.05 vs. EXO-mimic-NC group. ^#^*P* < 0.05 vs. EXO-si-NC group. ^&^*P* < 0.05 vs. EXO-miR-193a inhibitor + si-NC group. Measurement data were depicted as mean ± standard deviation, comparisons between two groups were assessed by *t-*test while those among multiple groups by one-way ANOVA followed with Tukey’s multiple comparisons test, the experiment was repeated three times.

Annexin V-FITC/PI double staining and Transwell assay indicated that the apoptosis rate of A549, A549/DDP, H1299, and H1299/DDP cells was enhanced while migration and invasion were impaired by miR-193a-overexpressed or LRRC1-downregulated BMSC-Exo. The apoptosis rate in A549, A549/DDP, H1299, and H1299/DDP cells was raised while migration and invasion were impaired by BMSC-Exo transfected with miR-193a inhibitor + si-LRRC1 by comparison with BMSC-Exo transfected with miR-193a inhibitor + si-NC. There was no obvious difference of the apoptosis rate, migration and invasion in cells treated with BMSC-Exo transfected with mimic NC, si-NC, or miR-193a inhibitor + si-LRRC1 (Fig. 7a–d).

The effect of BMSC-Exo with upregulated miR-193a on NSCLC cells in vivo suggested that miR-193a-overexpressed BMSC-Exo decreased tumor volume and weight after 4 weeks in contrast to mimic-NC-transfected BMSC-Exo (Fig. 7e, f).

## Discussion

Lung cancer is one of the most familiar diagnosed cancer, and one of the leading cause of cancer mortality around the world^24^. In a study conducted by Heller et al., it was shown that miR-193a was identified as a target gene for DNA methylation by genome-wide miRNA expression profiling in NSCLC^25^. It is customarily considered that LRRC1 is conducive to hepatocellular carcinoma (HCC) development and may be a potential target for the treatment HCC^17^. The current study was devised to explore the regulatory role of miR-193a/LRRC1 axis in affecting NSCLC DDP-resistance.

Our results supported the hypothesis that miR-193a expression was decreased and LRRC1 expression as enhanced in DDP-resistant NSCLC tissues and cells as well as miR-193a expression was correlated to TNM stage and differentiation degree of NSCLC patients. According to Wu et al., miR-193a expression is reduced in the development of dexamethasone resistance in myeloma cells^26^. In accordance with our finding, a recent study has presented that miR-193a expression is continuously reduced in NSCLC tissues^14^. Another study has presented that miR-193a expression is markedly declined in NSCLC tissues^27^. It is reported that LRRC1 expression is heightened in HCC samples in relation to adjacent non-cancerous livers^17^, which is in line with our findings. TNM stage is the main cancer staging system and the basic determinant of disease prognosis^28^. A study has purported that miR-193a-3p expression (the another product of pre-miR-193a) is depressed in NSCLC tissues, and the expression of miR-193a-3p is dramatically negatively associated with TNM stage^29^. Another study has indicated that miR-193a-3p expression is markedly reduced during the induction of osteoblast differentiation^30^. Moreover, it is revealed that miR-193a acts as a suppressive role in human BMSCs osteogenic differentiation^31^.

Two main results emerging from our data highlighted that BMSC-Exo reduced LRRC1 expression and heightened miR-193a expression. In addition, BMSC-Exo restrained the invasion, proliferation, and migration of drug-resistant cell in NSCLC and promoted cell apoptosis. It has been reported previously that the levels of urinary exosomal miR-193a are remarkably enhanced in children with primary focal segmental glomerulosclerosis compare to those in children with minimal change disease^32^. It is reported that the BMSCs-derived exosomes upregulating miR-16-5p restrains migration, proliferation, and invasion, while simultaneously induces the apoptosis of colorectal cancer cells in vitro^33^. Another study has verified that platelet-derived exosomes could also suppress the proliferation of PDGF-stimulated vascular smooth muscle cells^34^. It is displayed that placental stem cells derived from exosomes selectively restrain the growth of aggressive prostate cancer (PCa) cells^35^. Another study also demonstrated that TNF-α-induced exosomes could repress migration, tube formation, and boost endothelial apoptosis^36^. A prior research has generally confirmed that bone marrow stem cells-derived exosomes ameliorate osteoporosis via boosting osteoblast proliferation and repressing cell apoptosis^37^.

In addition, our study also suggested that BMSC-Exo with upregulated miR-193a and downregulated LRRC1 suppressed the colony formation, proliferation, invasion, and migration of NSCLC parent cells and drug-resistant cells. Moreover, BMSC-Exo with upregulated miR-193a reduced tumor volume and weight in NSCLC mice. It is reported that overexpression of miR-193a declines the colony formation and inhibits cell proliferation of NSCLC^13^. A study has shown that ectopic expression of miR-193a restrains colony formation, invasion, proliferation, and migration in A549 and H1299 cells, and overexpression of miR-193a reduces tumor xenografts growth in mice^38^. Ling et al. have reported that miR-193a suppresses PCa cell growth, migration, and invasion, as well as advances apoptosis in vitro. The in vivo results have indicated that upregulated miR-193a mediated by lentivirus dramatically declines PCa xenograft tumor growth^39^. It is revealed that downregulated LRRC1 suppresses the growth and colony formation of HCC cells^17^.

## Conclusion

To briefly conclude, our study suggests that BMSC-Exo shuffle miR-193a to suppress the colony formation, invasion, proliferation, and migration as well as advance apoptosis of NSCLC DDP-resistant cells via downregulating LRRC1. These findings provides a new insight in a novel target therapy for NSCLC. However, a conclusion about the effects of miR-193a/LRRC1 axis cannot be made explicitly due to the limited known researches on this. It needs to be surveyed rigorously and reported advisably in the future research.

## Supplementary information

supplement Figure 01
