# Supplementary figures and images for: Bone marrow mesenchymal stem cells-derived exosomal microRNA-193a reduces cisplatin resistance of non-small cell lung cancer cells via targeting LRRC1
Source: Cell Death Dis. 2020 Sep 25;11(9):801. doi: 10.1038/s41419-020-02962-4 (PMC7519084; doi:10.1038/s41419-020-02962-4)

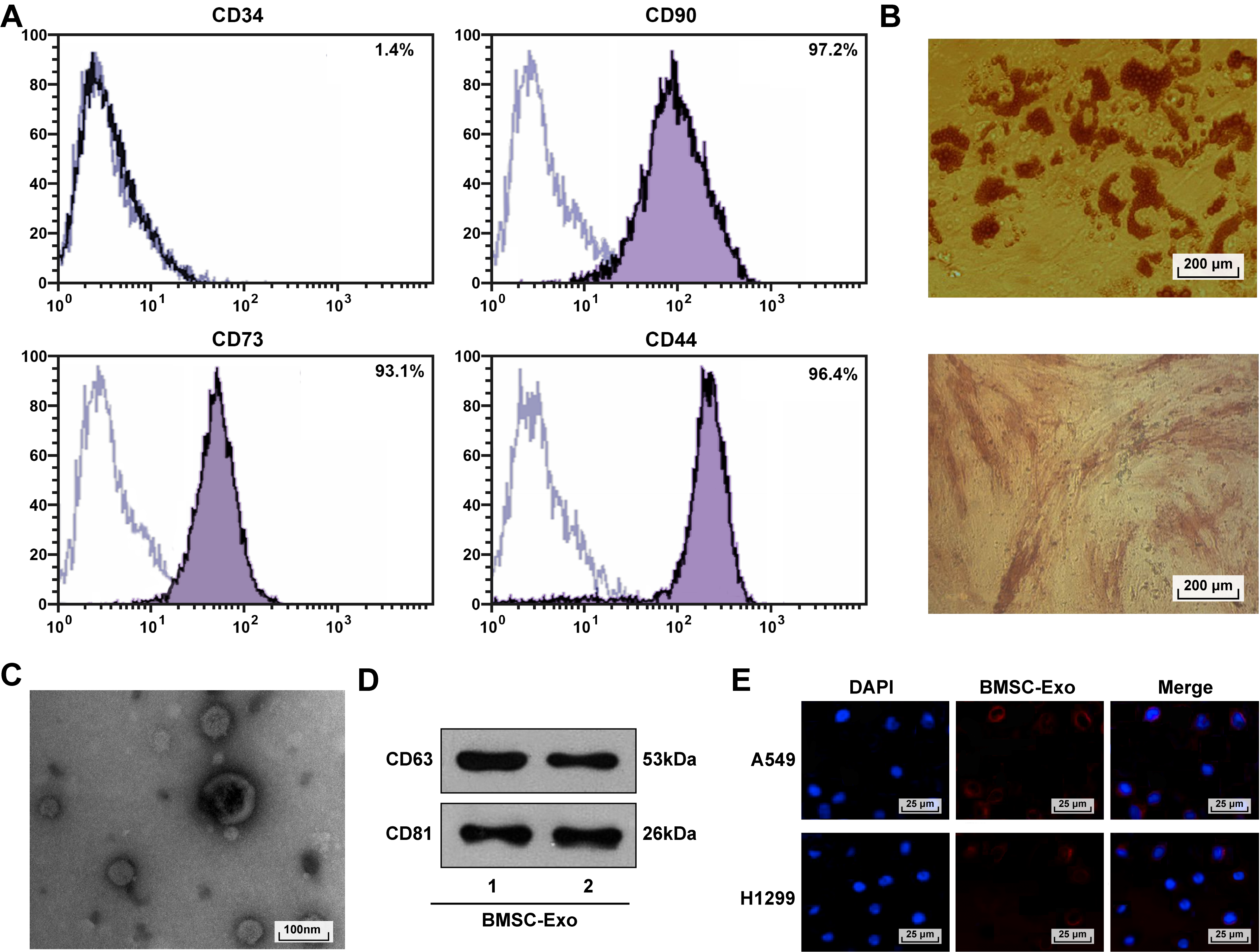

Supplement: Supplementary file 1 — supplement Figure 01 [file 41419_2020_2962_MOESM1_ESM.tif]
